# Rare Struma Ovarii Variant of Incidental Ovarian Dermoid Cyst in a Cadaveric Donor: A Case Report

**DOI:** 10.7759/cureus.102640

**Published:** 2026-01-30

**Authors:** Lauren Jernstadt, Nathaniel G Blanchard, Jenna Farnum, William McMillan, Paul Kowalski, Nicole L Geske, Libby J Bradley

**Affiliations:** 1 Anatomy, Michigan State University College of Osteopathic Medicine, East Lansing, USA; 2 Pathology, Michigan Pathology Specialists PC - Spectrum Health, Grand Rapids, USA

**Keywords:** abdominopelvic surgical procedure, fibrous adhesions, intrauterine pathology, ovarian dermoid cyst, post-hysterectomy

## Abstract

Post-operative adhesion is a common complication of abdominopelvic surgical procedures, including a hysterectomy. However, little is known about post-surgical tissue growths, atypical of fibrous adhesions. This case study evaluates a hysterectomized 80-year-old female anatomical donor who revealed intra-abdominal adhesions and bilateral abnormal tissue adnexal masses upon dissection and seeks to evaluate the causes of these abnormalities and potential consequences. Histologic analysis of the bilateral masses determined atrophied ovarian stromal tissue with a mature teratoma composed of respiratory epithelium and thyroid glandular tissue, consistent with struma ovarii, found in the left mass. Additional pathological findings included a proximally elongated rectum measuring 21.5 cm, a visible adhesion attaching a portion of sigmoid colon to the pelvic cavity wall, a section of sigmoid megacolon, and bilateral, smooth adipose herniations through the left and right superficial inguinal rings measuring 3.6 cm (left hernia) and 4.7 cm (right hernia). While intra-abdominal adhesions represent a major cause of post-operative complications following a hysterectomy, additional research is required on cases of teratoma complications by extensive pelvic fibrosis and the potential for active thyroid tissue in teratomas, causing death related to hypertension. The results of this case study contribute to the understanding of post-hysterectomy complications and incidental findings, aiming to do so through gross examination and histopathological analysis to better elucidate the etiology.

## Introduction

The female pelvic cavity houses vital structures related to various body systems. An understanding of this regional anatomy and the intimate relationships between the structures is crucial to healthcare professionals studying uro-renal, gastrointestinal, or reproductive systems [1]. Additionally, a variety of pathologic processes can have implications on both the structure and function of this region, examples ranging from diverticulitis, pelvic floor prolapse, to neurologic dysfunction [1]. The blood supply to female pelvic structures (bladder, uterus, and rectum) is primarily supplied by branches of the internal iliac artery, including the uterine, vaginal, superior vesical, and internal pudendal arteries, as well as the ovarian arteries, which typically arise directly from the abdominal aorta [2, 3]. In reference to the female anatomy significant in this case report, the uterus receives blood supply from the uterine artery and branches from the vaginal artery. The ovaries receive their blood supply from the ovarian arteries [2]. The rectum receives blood supply primarily from the superior rectal, middle rectal, and internal pudendal arteries [2]. The venous drainage from these structures largely parallels their arterial contributors.  

The uterus relies on many ligamentous attachments to support its central position within the pelvic cavity. These ligaments are the round ligament of the uterus, suspensory ligament of the ovary, cardinal ligament, broad ligament, uterosacral ligament, and ovarian ligament [4]. 

A procedure, such as a hysterectomy, can be required due to the diverse and broad pathologies concerning female pelvic anatomy. Some of these indications include malignancy, endometriosis, or irregular or heavy vaginal bleeding. There are also many elective indications like gender-confirming surgery, contraception, or cancer prophylaxis in families with known genetic predispositions [5, 6, 7]. 

There are varying degrees of a hysterectomy, each of which may have its own specific indications and approach based on both the patient’s condition and the required degree of removal. These procedures range from subtotal (a supracervical excision), total or “simple” (removal of uterus and cervix), or radical (removal of uterus and cervix, including the parametrium and upper vagina). Additional features could include salpingectomy or oophorectomy, depending on the circumstances. Radical hysterectomy can be further classified by the degree and location of excision involving the paracervix, lateral parametrium, ventral parametrium, or dorsal parametrium [8]. 

The complications of a hysterectomy much resemble those of many other intra-abdominal surgical procedures. Complications like adhesions, strictures, and fistulas are all common. More commonly, there is a risk of infection, nerve injury, and damage to the gastrointestinal or genitourinary tracts [9]. Pelvic masses following a hysterectomy may also be observed [10]. Deciding not to excise the ovaries and uterine tubes during a hysterectomy may increase the risk of forming ovarian cysts or other related malignancies [10]. One possible complication is physiological cysts or teratomas, especially in patients with a history of adenomyosis or endometriosis [10]. Mature cystic teratomas, or alternatively an ovarian dermoid cyst, are one of the most common benign ovarian masses. Most often, these masses are found incidentally, due to their common asymptomatic nature. A dermoid cyst is characterized by the presence of ecto-, endo-, or mesodermal tissue within it, presenting as teeth, skin, hair, muscle, or thyroid tissue [11]. 

Both patients and physicians must be aware of the wide range of complications that could arise due to a hysterectomy, to take both preventative measures and encourage follow-up imaging to identify any warning signs promptly to avoid further dysfunction. 

## Case presentation

**Figure 1:**
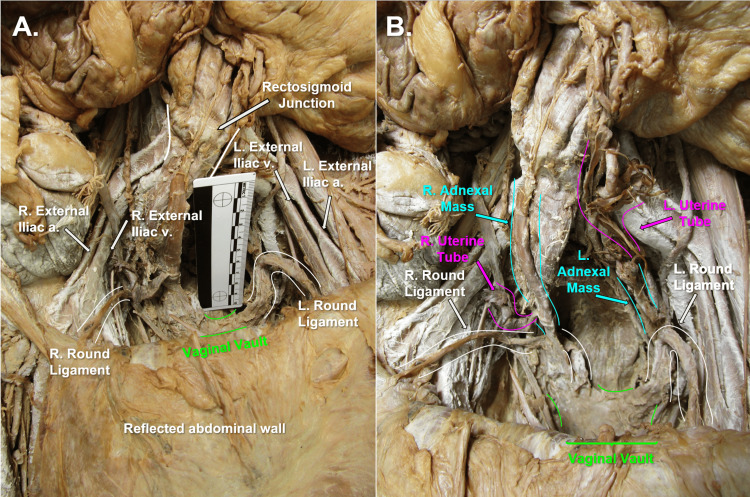
Donor Abdominopelvic Cavity, Anterior View Panel A) Anterior view of donor abdominopelvic cavity with anterior abdominal wall reflected and vaginal vault highlighted in green. Panel B) Anterior view of donor abdominopelvic cavity with highlighted right and left round ligaments in white, uterine tubes highlighted in pink, adnexal masses highlighted in blue, and vaginal vault highlighted in green.

**Figure 2:**
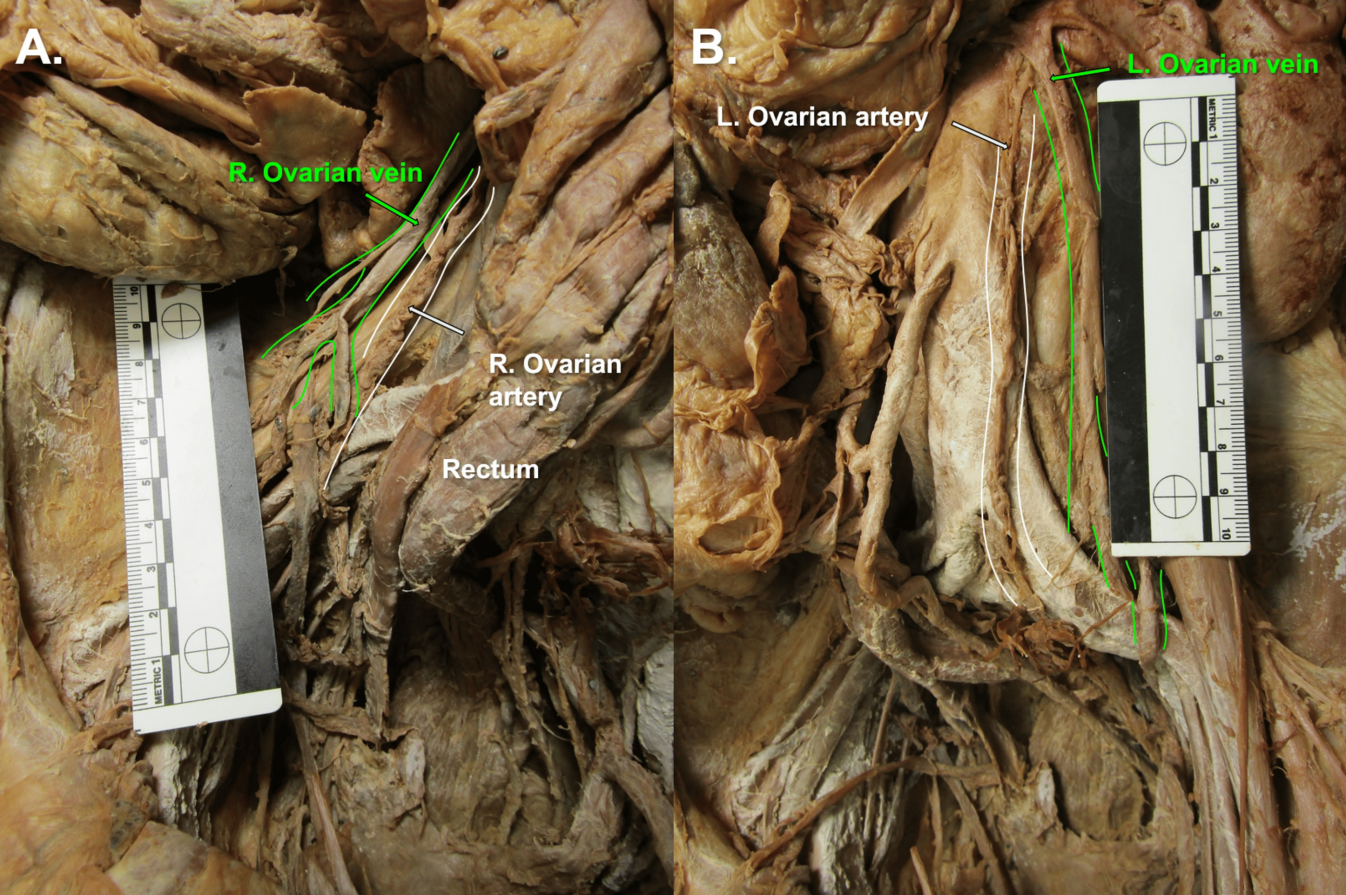
Tortuous Ovarian Arteries (Highlighted in White) And Distally Branched Ovarian Veins (Highlighted in Green) Panel A) Right Ovarian Artery and Vein. Panel B) Left Ovarian Artery and Vein.

**Figure 3:**
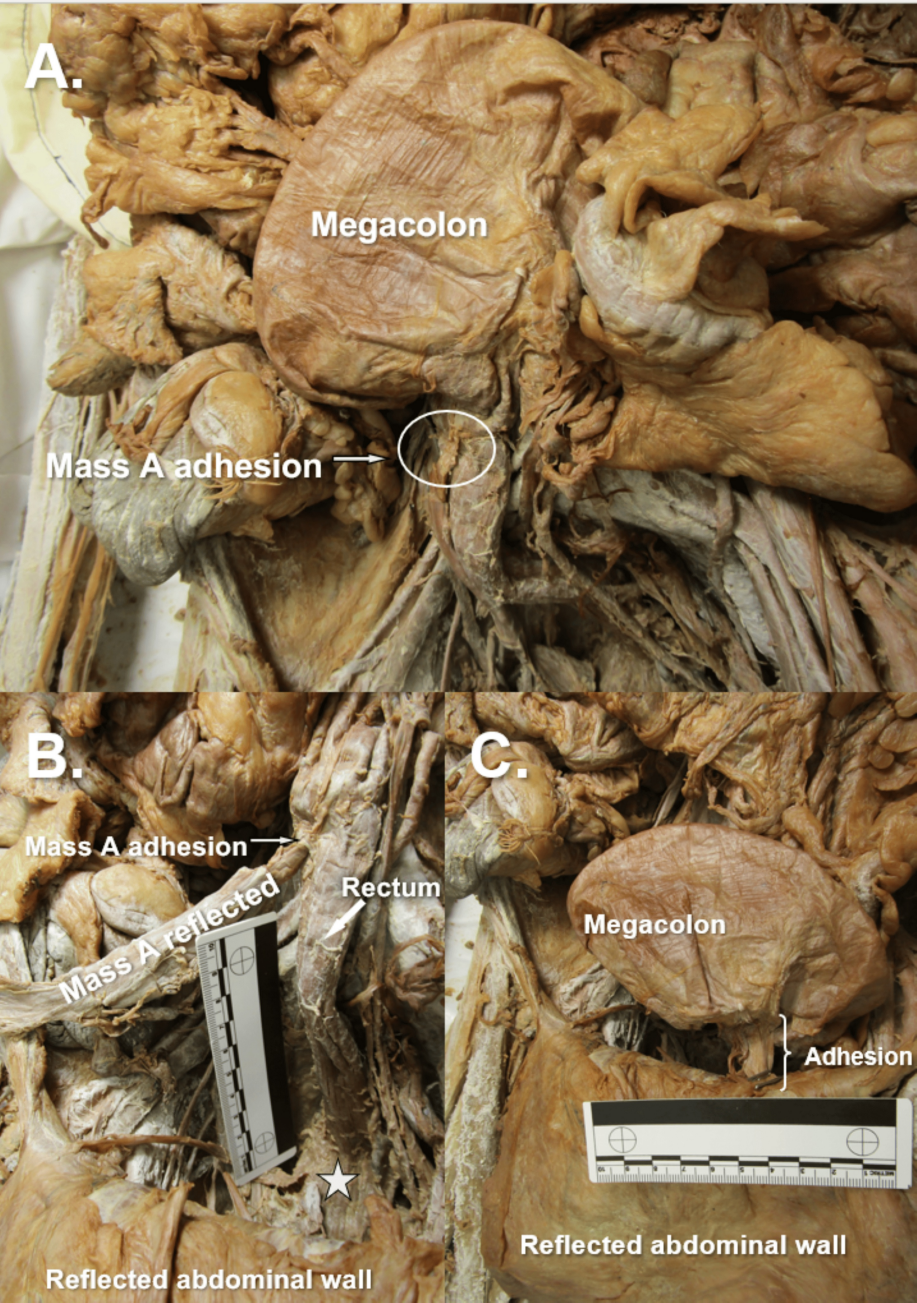
Gastrointestinal Findings and Adhesions Panel A) Anterior view of the distal gastrointestinal tract showcasing sigmoid megacolon and the attachment point of adnexal mass A at the rectosigmoidal junction. Panel B) Mass A reflected to showcase proximal elongation of the rectum with a star located at the distal end of the rectal canal. Panel C) Sigmoid megacolon showcased with adhesion attaching to the anterolateral abdominopelvic cavity wall.

**Figure 4:**
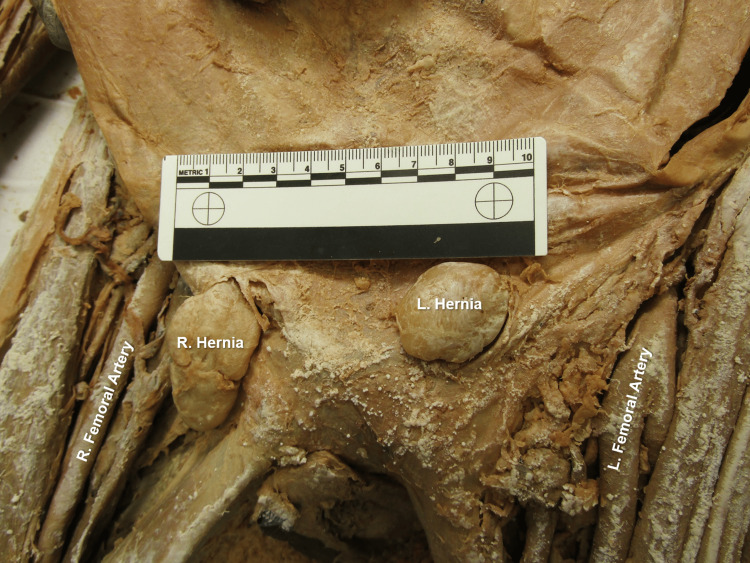
Bilateral Inguinal Hernias Bilateral indirect inguinal hernias extruding through superficial inguinal rings.

Immediate dissection of an 80-year-old female anatomical donor's abdominopelvic cavity, involving removal of the jejunum and ileum, and improved visualization of the neurovasculature throughout the abdominopelvic cavity, revealed gross evidence of a hysterectomy (Figure 1A). Bilateral uterine tubes and bilateral abnormal adnexal masses were visible, and bilateral round ligaments of the uterus attached proximally to the respective ipsilateral superolateral poles of the vaginal vault (Figure 1B). Multiple anatomical abnormalities were discovered, including bilateral tortuous ovarian arteries (Figure 2), bilateral ovarian veins with multiple branches at the distal ends (Figure 3), sigmoid megacolon (Figure 3A), and bilateral inguinal fat hernias (Figure 4). An adhesion band was found attaching a portion of sigmoid colon to the left anterolateral pelvic wall (Figure 3C), with megacolon persisting approximately 15 cm distal to the adhesion until the rectosigmoidal junction.

**Figure 5:**
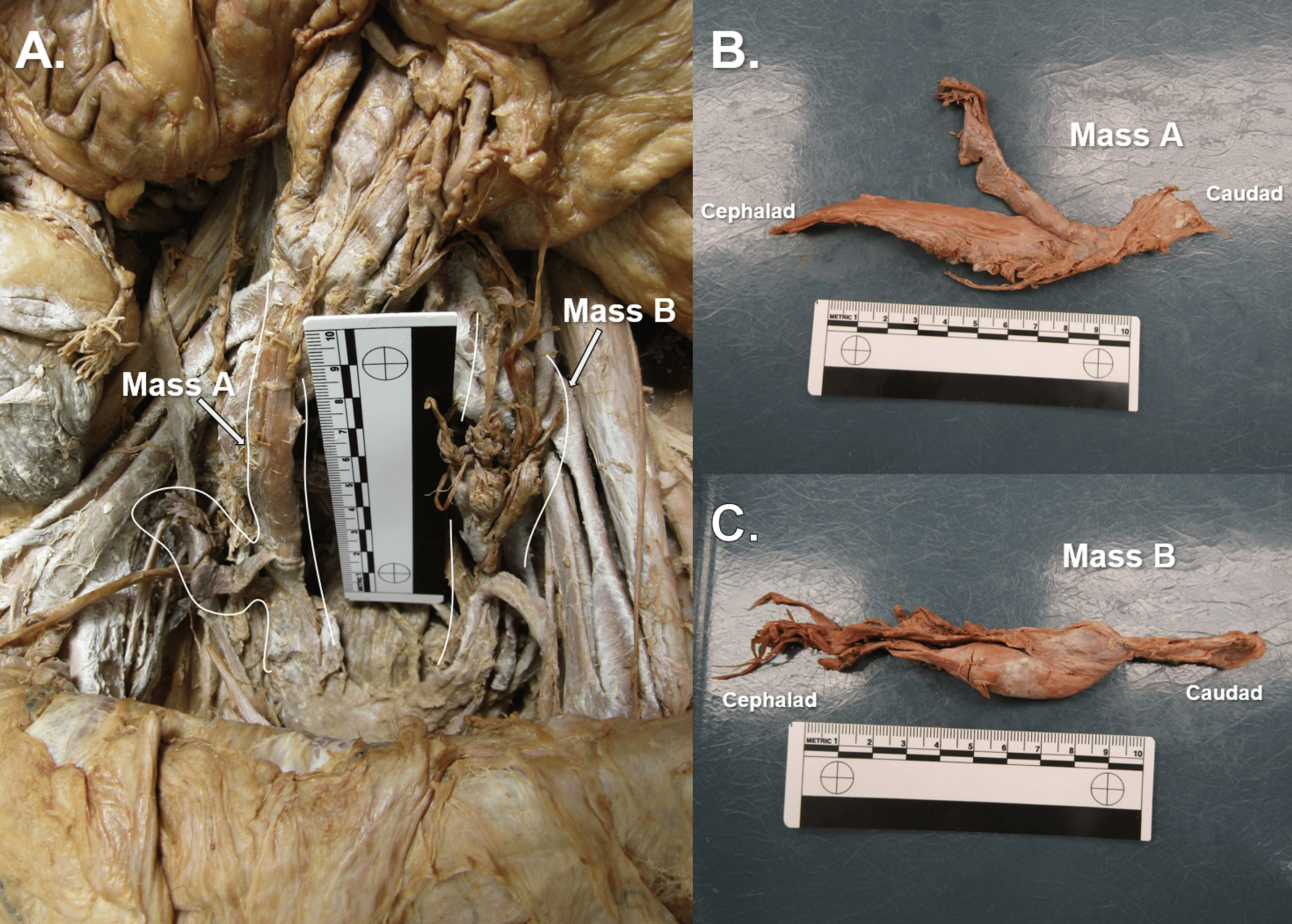
Pelvic Masses A and B Panel A) Anterior view of donor abdominopelvic cavity highlighting right (Mass A) and left (Mass B) adnexal masses. Panel B) Mass A excised. Panel C) Mass B excised.

The bilateral adnexal masses were fairly symmetric in shape, presenting as oblong and slim structures measuring approximately 10.7 cm (right adnexal mass, Mass A) and 5.1 cm (left adnexal mass, Mass B) in length (Figure 5). Both masses had similar texture upon palpation, feeling solid and firm. Mass A was found with distal attachments at the joining of the proximal right round ligament of the uterus to the right superolateral pole of the vaginal vault; the proximal attachment of the right adnexal mass via a fibrous band located at the rectosigmoidal junction, just distal to the end of the megacolon segment. Mass B was revealed to the authors after the adhesion connecting the sigmoid colon to the left anterolateral pelvic wall was severed, presenting a mass with distal attachment along the left round ligament of the uterus at its proximal end. Retained uterine tubes were found attached to the distal ends of both adnexal masses.

The donor’s rectum was found proximally elongated and measuring approximately 21.5 cm in length (Figure 3B). Rectal tissue was grossly striated, and a diverticulum was appreciated at the caudal-most portion of the rectum within the pelvic cavity. The right adnexal mass fibrous band attachment is seen at the rectosigmoidal junction and corresponds to the distal end of the sigmoid megacolon section (Figure 3B). These findings, along with the adhesion of the sigmoid colon to the pelvic wall, could suggest a state of increased intra-abdominal pressure as evidenced by the bilateral fatty inguinal hernias and superior displacement of abdominal cavity organs [12].

Histopathology

**Figure 6:**
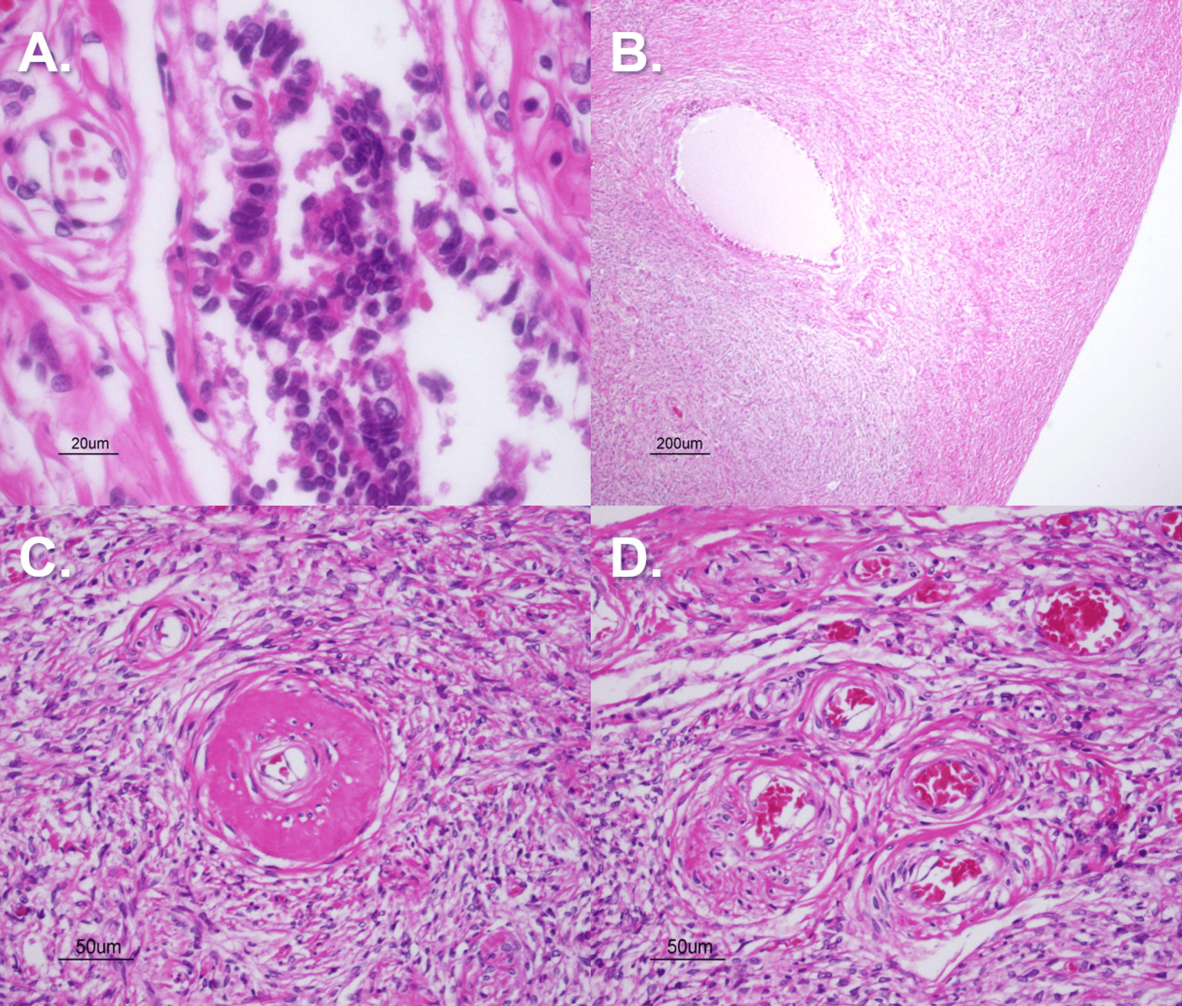
Pelvic Cavity Mass A Pathological Findings Panel A) Right Uterine Tube– Fibrotic peritubal tissue (H&E stain; 40x magnification). Panel B) Right Ovary Ovarian Stroma– hyalinized ovarian stroma (H&E stain; 4x magnification). Panel C) Hyaline arteriolosclerosis present in ovarian stroma (H&E stain; 20x magnification). Panel D) Additional ovarian vasculature and surrounding involuting stroma (H&E stain; 20x magnification).

**Figure 7:**
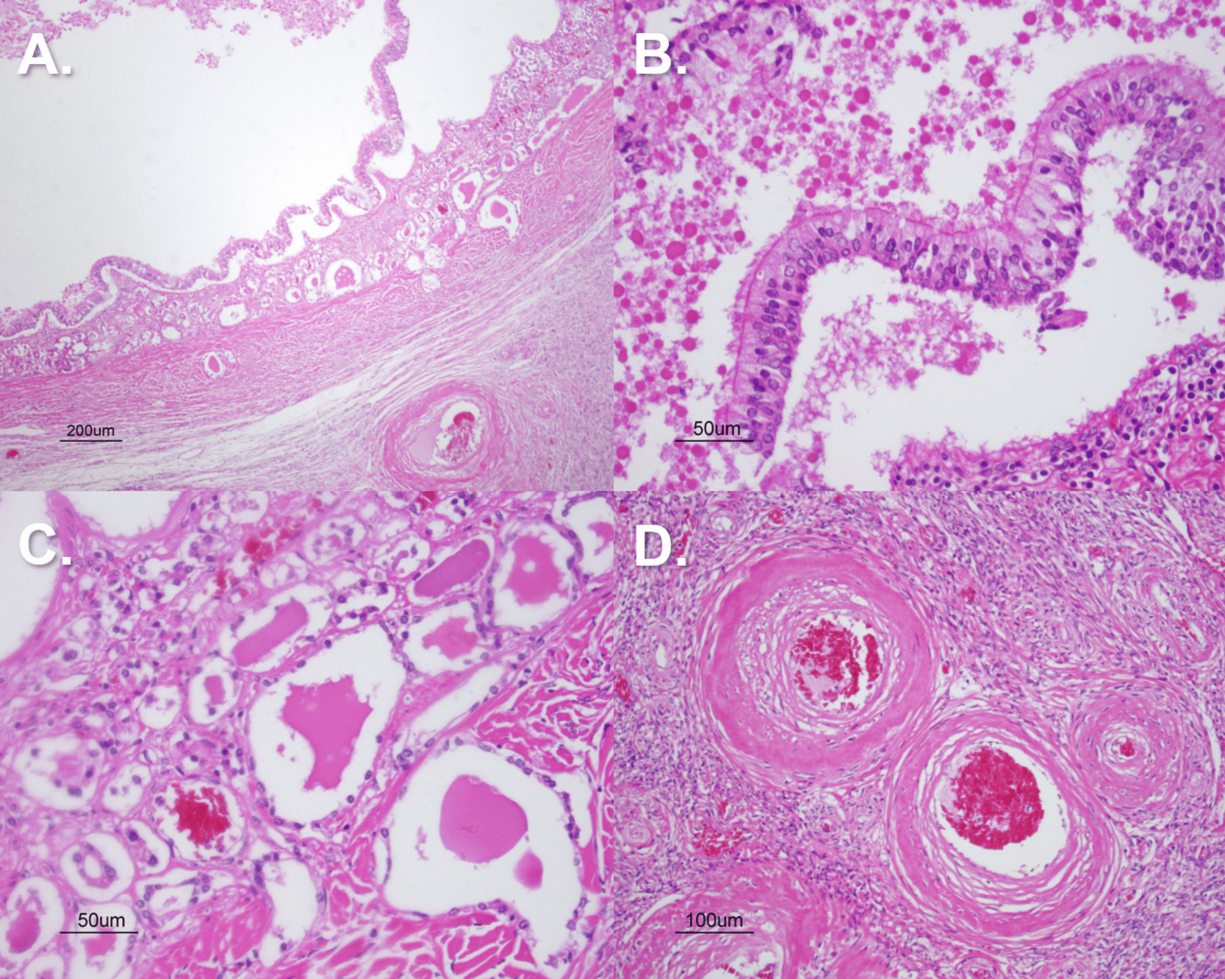
Pelvic Cavity Mass B Pathological Findings Panel A) H&E stain reflecting a cross-section of dermoid cyst (4x magnification). Panel B) H&E stain reflecting respiratory epithelium within the dermoid cyst (20x magnification).  Panel C) H&E stain indicating mature thyroid tissue and follicles containing eosinophilic colloid (20x magnification). Panel D) H&E stain indicating hyperplastic and hyaline changes noted within vessels of the mass (10x magnification).

To investigate possible pathology underlying the adnexal tissue masses, sections of tissue were taken from both adnexal masses along with the right uterine tube and were stained with hematoxylin and eosin (H&E) stain. Histology of Mass A was consistent with ovarian involution with atrophic, hyalinized ovarian stroma throughout the section (Figure 6). Mass B histology showed similar findings of ovarian stroma throughout but also exhibited features of a small teratoma throughout the sections, as evidenced by the epithelium-lined glands filled with hyaline-like “colloid” secretions suggestive of thyroid gland tissue and pseudocolumnar epithelium with microvilli projections throughout suggestive of lung tissue. Specifically, the lack of spacing between microvilli led the authors to believe that the tissue was not uterine tube in nature, as a section of the right uterine tube revealed the “tufts” of microvillous projections spaced every five to eight cells apart, which is characteristic of uterine tube epithelial lining. Vasculature throughout sections of the left adnexal mass revealed a combination of thick hyalinized vessel walls, pathognomonic of non-malignant hypertension, and “onion skinning” of the endothelium, pathognomonic of malignant hypertension. These vasculature changes could very likely be due to a state of generalized hypertension in our donor, which could be possible given the history of stroke and cerebral infarction (Figure 7). A section of the abdominal adhesion connecting the sigmoid colon to the abdominopelvic cavity wall was also stained with H&E, and findings of hyaline connective tissue, minimal vascularization, and adipose tissue were consistent with the histologic presentation of a fibrous abdominal adhesion.

## Discussion

Gross and histologic evaluation provide compelling evidence for the diagnosis of a mature cystic teratoma - commonly referred to as a dermoid cyst - occurring in the setting of widespread pelvic fibrosis. The authors propose that these represent two distinct pathological processes occurring concurrently. This hypothesis is supported by the unilateral left ovarian involvement, which is consistent with the localized presentation typical of teratomas [13].

Mature cystic teratomas are composed of well-differentiated elements derived from all three germ layers. Unlike hamartomas, teratomas contain tissues that are not native to their site of origin [14]. As illustrated in image 6A, low-power magnification reveals a well-defined cystic lesion embedded within ovarian stroma. High-power examination, in image 6B, demonstrates pseudostratified ciliated columnar epithelium with interspersed goblet cells, an epithelial configuration indicative of respiratory tract differentiation [15]. 

Further supporting the diagnosis is the identification of mature thyroid tissue within the lesion. This finding is consistent with struma ovarii, a rare but recognized monodermal variant of mature cystic teratoma [16]. In image 6C, thyroid tissue appears at the interface of the cyst and surrounding ovarian stroma, exhibiting well-formed follicles containing eosinophilic colloid material.

Although the patient’s clinical history is limited, the presence of thyroid tissue raises the possibility of a hormonally active struma ovarii. If functional, this ectopic thyroid tissue may have contributed to a state of hypertension in the patient [17]. Additional histologic evidence supportive of chronic hypertension includes widespread arteriolosclerosis. Numerous vessels exhibit concentric wall thickening with features of both hyperplastic and hyaline change, as seen in 6D. The authors speculate that these vascular findings may have contributed to the patient’s suspected cerebrovascular event and the ultimate cause of death.

The presence of fibrotic tissue throughout the pelvic cavity is consistent with extensive adhesion formation. Adhesions are a well-documented postoperative complication, representing an aberrant wound-healing response in which fibrous bands form abnormal connections between intra-abdominal organs or surfaces [18]. Their pathogenesis is closely associated with mesothelial cell injury, which can be precipitated by infection, trauma, or prior surgical procedures [19]. In this case, the patient’s history of total hysterectomy serves as a plausible inciting event for the development of pelvic adhesions. As noted by Tabibian et al. [20], adhesions may result in serious clinical complications, including bowel obstruction. The authors propose that the megacolon observed on gross examination likely developed secondary to adhesive bands located distal to the dilated colonic segment, resulting in functional obstruction and significant colonic enlargement.

It should be noted that the authors were provided with a very limited clinical and surgical history of the donor, and that there is a potential that the donor underwent other abdominopelvic surgeries outside of a hysterectomy that could have contributed to the gross findings in the donor's pelvic cavity.

## Conclusions

This case report contributes to the limited body of literature on female pelvic teratomas and presents a unique case complicated by underlying pelvic fibrosis following a hysterectomy. The authors propose a reasoned and evidence-based explanation for the patient’s cause of death, aiming to prompt clinicians to consider rare but clinically significant diagnoses in patients presenting with similar symptoms. By highlighting this unusual presentation, the report seeks to raise awareness and provide a relevant clinical example to support future diagnostic decision-making.
